# Developing a tool for mapping adult mental health care provision in Europe: the REMAST research protocol and its contribution to better integrated care

**DOI:** 10.5334/ijic.2417

**Published:** 2015-12-01

**Authors:** Luis Salvador-Carulla, Francesco Amaddeo, Mencia R. Gutiérrez-Colosía, Damiano Salazzari, Juan Luis Gonzalez-Caballero, Ilaria Montagni, Federico Tedeschi, Gaia Cetrano, Karine Chevreul, Jorid Kalseth, Gisela Hagmair, Christa Straßmayr, A-La Park, Raluca Sfetcu, Kristian Wahlbeck, Carlos Garcia-Alonso

**Affiliations:** Mental Health Policy Unit, Brain and Mind Centre, Centre for Disability Research and Policy, Faculty of Health Sciences, The University of Sydney, Camperdown, Australia; Section of Psychiatry, Department of Public Health and Community Medicine, University of Verona, Verona, Italy; PSICOST Research Association, University of Loyola Sevilla, Sevilla, Spain; Section of Psychiatry, Department of Public Health and Community Medicine, University of Verona, Verona, Italy; Department of Statistics and Operations Research, University of Cadiz, Cádiz, Spain; Section of Psychiatry, Department of Public Health and Community Medicine, University of Verona, Italy; Department of Neuroepidemiology, University of Bordeaux, Talence, France; INSERM, U897, Neuroepidemiology Team, F-33000, Bordeaux, France; Section of Psychiatry, Department of Public Health and Community Medicine, University of Verona, Verona, Italy; Social Care Workforce Research Unit, King's College London, London, UK; Section of Psychiatry, Department of Public Health and Community Medicine, University of Verona, Verona, Italy; Université Paris Diderot, Sorbonne, Inserm, ECEVE, U1123, F-75 010; AP-HP, URC-Eco, Paris, France; Department of Health Research, SINTEF Technology and Society, Trondheim, Norway; IMEHPS.Research, Vienna, Austria; IMEHPS.Research, Vienna, Austria; Personal Social Services Research Unit, LSE Health and Social Care, London School of Economics and Political Science, London, UK; Institute for Economic Forecasting Bucharest, Romania; Department of Mental Health, National Institute for Health and Welfare (THL), Helsinki, Finland; Vice-chancellor for research, Universidad Loyola Andalucía, Sevilla, Spain; For the REFINEMENT GROUP

**Keywords:** mental health care, evidence-informed policy, health service research, integrated care

## Abstract

**Introduction:**

Mental health care is a critical area to better understand integrated care and to pilot the different components of the integrated care model. However, there is an urgent need for better tools to compare and understand the context of integrated mental health care in Europe.

**Method:**

The REMAST tool (REFINEMENT MApping Services Tool) combines a series of standardised health service research instruments and geographical information systems (GIS) to develop local atlases of mental health care from the perspective of horizontal and vertical integrated care. It contains five main sections: (a) Population Data; (b) the Verona Socio-economic Status (SES) Index; (c) the Mental Health System Checklist; (d) the Mental Health Services Inventory using the DESDE-LTC instrument; and (e) Geographical Data.

**Expected results:**

The REMAST tool facilitates context analysis in mental health by providing the comparative rates of mental health service provision according to the availability of main types of care; care placement capacity; workforce capacity; and geographical accessibility to services in the local areas in eight study areas in Austria, England, Finland, France, Italy, Norway, Romania and Spain.

**Discussion:**

The outcomes of this project will facilitate cooperative work and knowledge transfer on mental health care to the different agencies involved in mental health planning and provision. This project would improve the information to users and society on the available resources for mental health care and system thinking at the local level by the different stakeholders. The techniques used in this project and the knowledge generated could eventually be transferred to the mapping of other fields of integrated care.

## Introduction

Community mental health care is a relevant precursor of integrated care [1]. It started in the 1960s in the UK and the USA and spread to the majority of Western countries by the mid 1980s following the deinstitutionalisation process and closure of old mental hospitals. This model was first implemented in the UK and several jurisdictions in the USA and The Netherlands, followed by the Scandinavian countries, and Southern European countries such as Italy and Spain. Although significant differences existed across these approaches, a number of commonalities anticipated the development of the integrated care approach in the 1990s. The model of community psychiatry emphasised an integrated delivery system encompassing horizontal integration of the specialised community mental health system and vertical integration of this secondary care with hospital or tertiary care as well as with primary care. The horizontal integration included the deployment and coordination of outpatient community mental health centres with other health services – residential alternatives to hospitalisation in the community, day hospitals and day care rehabilitation services – and other types of social day care, education, employment and supported accommodation [2–4]. The organisational components of the community care model in mental health also included high-intensity coordination as in the Assertive Community Care model [2], development of multidisciplinary teams including case managers. However, these early contributions were not accompanied with the completion of a fully integrated care system and an efficient monitoring. On the contrary, major problems persist 30 years after the community mental health care system was implemented.

The recent European Health Action Plan [5] and the WHO Mental Health Gap Action Programme (mhGAP) [6] indicate that there is a gap between service provision and the needs of persons experiencing mental health problems. This gap has been well documented in different mental health conditions by the analysis of perceived needs in relation to service utilisation [7] or the combined analysis of epidemiological data and standard description of service provision and utilisation at local [8] and national levels [9], particularly in complex cases with physical comorbidities [10,11].

More coordination is required to enhance local community-based services organised around the needs of a population catchment area in an integrated care system. The assessment and monitoring of the transformation of the system should follow multi-step evaluation strategy, as the one followed in monitoring the plan of mental health in Catalonia (Spain) [12]. The first step of this overarching evaluation should include the mapping of the existing services in order to provide a snapshot of the availability and capacity of the care system, to then analyse the coordination networks, the financing flows and the relationship of these components with the use of the available services, the culture or philosophy of care and the efficiency system [13] from an integrated care perspective.

The mapping of services is the first step in this building-block strategy and it should use internationally validated tools that could provide a comprehensive and systematic description of all the mental health resources available in a local area, the utilisation of these resources and the comparability with other jurisdictions. As a matter of fact, the comparative information on the European Union mental health local and regional systems is surprisingly low, most of it being limited either to the description of specific types of services or to the collection of general indicators that can hardly be used for actual priority setting and resource allocation.

This lack of regional comparison could be related to several factors [9,14]: (1) the complexity of the mental health system that hampers international comparison; (2) the difficulty of agreeing upon comparable units of analysis at macro- (countries, regions), meso- (catchment health areas) and micro-levels (individual services); (3) the wide variability in the terminology of services and programmes even in the same geographical area and the low usability of listings of services by their names (e.g., day hospitals, day centres, social clubs, etc.), as the service name could not reflect the actual activity performed in the setting; (4) the low number of service assessment instruments that have undergone a full validation process and proven its usability in independent studies and in actual policy planning.

The REMAST study is part of the EU-funded project REFINEMENT (REsearch on FINancing systems’ Effect on the quality of MENTal health care), which is aimed at mapping and describing the characteristics of financing systems for mental health care in eight European countries, using detailed descriptions of eight mental health systems, their service provision, care pathways and quality. REMAST aims at developing a new decision tool (REFINEMENT MApping Services Tool) by combining a series of standardised health service research instruments and Geographical Information Systems (GIS) for developing local atlases that may facilitate the monitoring, reviewing and improving of mental health systems in Europe. The REMAST objectives are: (1) to describe the existing services for people experiencing mental disorders in eight regions in Europe, (2) to compare the availability of resources and the care capacity across the different regions; and (3) to assess the accessibility of services in the eight catchment areas. This basic information of the local context is crucial to evaluate, analyse and monitor the development of integrated care.

## Methods

### Study design

This context analysis follows an ecological design which combines knowledge discovery (standard description of service provision) and implementation (use for health planning). It has been carried out in eight European Union countries that encompass cases of different mental health care systems in Europe including Scandinavia, Central Europe, UK, Mediterranean and Eastern European countries. Nine countries participated in the general REFINEMENT project: Austria, England, Estonia, Finland, France, Italy, Norway, Romania and Spain (Catalonia). Data from Estonia could not be double checked and were incomplete so this country was not included in the REMAST study.

### Units of analysis and inclusion/exclusion criteria

It is important to note that there are different units of analysis in health care and that comparisons must be made across the same unit to prevent the incommensurability bias [15]. The lack of comparisons like-with-like has been previously identified as one of the major problems in health service research [16]. In most service research studies, “services” and “interventions” are taken as the “unit” of comparison. However, these are complex constructs where several other units of care provision can be identified: main types of care, care modalities, care programmes, care packages, single interventions, activities and even the philosophy of care [16]. Furthermore comparison can be made across different catchment areas, meso-organisations within a catchment area (e.g., hospitals) or macro-organisations (e.g., large health care corporations providing care in several areas).

The standard description of the local mental health system in REMAST was based on a modification of the mental health matrix [17]. This matrix arranges the indicators of the mental health system according to the Donabedian model of the production of health care [18] (inputs, throughputs and outputs) at three levels: macro (country, region); meso (health district, small health area); and micro (individual care). In REMAST, “services” instead of individual care where regarded at the micro-level.

To allow for inter-territorial comparison of service provision at meso- and at micro-levels, different units of analysis have been taken into account in this study. At the meso-level, we used defined local areas. At the micro-level, we used three different units of analysis of the micro-organisation of care.

### Meso-level: study areas (study location and selection criteria)

Eight catchment “study” areas were selected in the eight countries according to a population size between 200,000 and 1,500,000 inhabitants, and coverage of at least one health district which was not limited to a macro-urban area within a municipality (e.g., the study was not focused in cities such as Helsinki, Paris or Barcelona). Additional characteristics considered in the selection of study areas were the availability of appropriate sources of information on the local Mental Health System (MHS), the previous knowledge of the data sets, and cooperation with local, regional and national health officers in Finland and Spain.

The geographical description included different administrative boundaries such as municipalities, census areas, health districts and small mental health areas depending on community mental health centres where available. The health zoning subtypes described at the DESDE-LTC service classification system were used for mapping the “sectorisation” or catchment areas served by a hospital (H3), a mental health care centre (H4) or a primary health care centre (H5) (see www.edesdeproject.eu for further information). The rates of availability, capacity and utilisation were calculated in relation to the population assisted by every service in their corresponding catchment areas.

The study areas selected were: Industrieviertel (Federal State of Lower Austria); Hampshire including Portsmouth and Southampton Unitary Authorities (England); Helsinki and Uusimaa Hospital District (Finland); Loiret Department with seven sectors of psychiatry of the Georges Daumézon hospital (France); Verona Mental health Department (Veneto, Italy); Sør-Trøndelag (Norway); Jud Suceava (Romania); and Girona Health District (Catalonia, Spain). Geographical size varied from 1061 km^2^ in Italy to 18,856 km^2^ in Norway. The detailed characteristics of the catchment areas are available at the REFINEMENT webpage (www.refinementproject.eu).

### Micro-level (units of analysis of the micro-organisation of care)

Three different units of analysis of the micro-organisation of care were considered in this study.

#### Services described by their names

This refers to the traditional category of “service” as the standard organisation of care delivery at micro-level available in every area (e.g., acute ward, day hospital, community mental health centre, etc.). All the mental health care services provided at the local areas and defined by their names were identified and described. In this study we included all services with publicly funded care and universal access providing direct mental health and “other care” (e.g., social, employment, training) to adults (+18) with a psychiatric disorder which were available for the study areas during the year 2010. We excluded specialised services for persons with alcohol and drug dependence, persons with intellectual disabilities, child and adolescents, and criminal justice. All services especially dedicated to the treatment of the elderly (e.g., nursing homes, mobile home nursing services, etc.), unless they provide services especially for people with mental disorders, were also excluded.

“Direct mental health care” included all specialised outpatient, day and residential services. As to get a complete overview of the services available for the residents, those mental health services which were located outside the study area but which served the patients of the study area were also counted. General services providing care for MH patients were described and coded (e.g., generic medical acute wards that admitted psychiatric patients in a routine basis, and primary care centres) but they were not included in the REMAST comparison analysis unless they had at least 20% of users with mental disorders.

#### Basic units of production of care (Basic stable inputs of care - *BSICs*)

Basic stable inputs of care are the minimal stable units of production of care or “stable functional teams” that can be described in every service. They are identified by their temporal and organisational stability defined by their placement, target patients, common staff and administrative autonomy. On some occasions it is difficult to differentiate the basic units of production of care from simple activities or from clinical programmes and packages of care delivered at the service. A series of inclusion criteria have been designed and tested in previous studies for that purpose [9,16]. The definition of BSIC and its selection criteria are described in Table 1.

#### Main types of care (MTCs)

**Figure 1.**
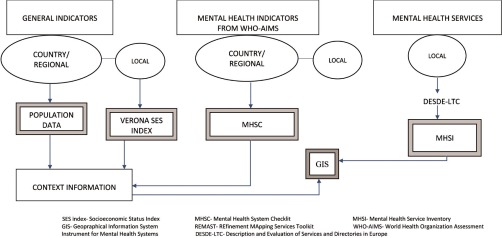
Visual representation of the components of the REMAST toolkit.

In order to classify the basic stable inputs of care identified in a service and in a given jurisdiction, a smaller unit of analysis was developed in previous studies on ESMS/DESDE [16]. The “main type of care” (MTC) is a classification system of BSICs comprising over 90 codes. These codes have been developed following a bottom-up approach based on the description of actual services in over 10 countries in Europe in a succession of mental health service research studies carried out by the Evaluation of Psychiatric Care Assessment Team (EPCAT) group. The MTC identifies the more meaningful care structure or activity offered by the BSIC that allows clustering similar units of production of care and differentiating them from other types of care. Once a BSIC has been identified it is labelled according to a number of descriptors (types and qualifiers), such as status of user, care typology, intensity, time of stay and mobility. Typically, a BSIC could be described by a single MTC, but in some cases it is necessary to include a principal main type (e.g. a “residential” code) and an additional one (e.g., a “day care” code). In previous studies, over 70% of the units of production of care in different jurisdictions were described with a single MTC, and very rarely a unit needed more than four codes to be fully described. The formal definition of MTC is provided in Table 1, and the DESDE-LTC taxonomy tree is shown in Figure 1. The complete “main types of care” coding system can be consulted elsewhere [16]. The DESDE-LTC taxonomy tree is shown in Figure 1.

Ideally, a service corresponds to one BSIC that could be described by one MTC, but this is not always the case as it could be expected from the complexity of services and health systems. Usually, the services identified by their names in a jurisdiction are composed by a larger number of BSICs, and these BSICs require a higher number of MTCs to provide a meaningful description of the local care system that could be effectively represented in a geographical information system.

### Material

The REMAST tool is compounded of a battery of checklists/inventories, instruments and indexes. Its modular structure allows for future incorporation of other components relevant for mental health system assessment. It contains five main sections: (a) Population Data; (b) Verona SES Index [19]; (c) Mental Health System Checklist describing policies and organisation of mental health care through selected WHO-AIMS 2.2 items [20,21]; (d) the Mental Health Services Inventory allowing the mental health services of a selected study area to be classified according to the ESMS/DESDE approach [1,14] using the DESDE-LTC instrument for providing detailed descriptions of the coding and characteristics of services from all relevant sectors (health, social, education, employment, housing and justice), including their utilisation and staffing [16]; and (e) Geographical Data: the description of geographical context of the study area. A visual representation of the components of REMAST toolkit is depicted in Figure 1.

### Population data

In order to provide a socio-demographic description of both the study and the macro-areas, the number of inhabitants is requested for each area by distinguishing males and females in different age groups (total population: 0–17 years; 18–64 years; 65 years and older). The reference year of the available data is also required together with the nomenclature of territorial units for statistics (NUTS) code when applicable.

### Verona SES index

The socio-economic status [22] deprivation index is an indicator of the resources individuals, households, groups or areas have at their disposal and which affect their power and life opportunities. The construction of such an indicator may vary across areas and time periods, depending on which variables affect social ranking in the society [19]. The variables include family composition (single-parent families, individuals divorced or widowed), employment (workers employed in industry and services respectively, unemployment rate), education (individuals with tertiary qualification on people aged 15 or older), household size (average number of people per household, households made up by 1 person and by 5 or more people) and age composition (ageing index, dependency ratio, individuals below 5 years old). Other variables (rented accommodation, population density and immigrants) were also included.

### Mental health system checklist (MHSC)

General information on Mental Health Policy; Mental Health Plan; Monitoring and Training on Human Rights; and Organizational Integration of Mental Health Services are provided through the administration of 10 selected items from the WHO-AIMS 2.2 [20,21]. Data are requested at local, regional and national level. Generally, all information should be available through institutions at national or regional level but some facilities are to be contacted to get data directly from them. The organisation of the mental health system is described with a focus on the integration of mental health services and communities, and the innovation in legislation for mental health care.

### Mental health service inventory (MHSI)

The Mental Health Services Inventory section is aimed at describing all Mental Health Care services of the study area providing health and social care to people with a psychiatric disorder. When possible, so as to get a complete overview of the services available for the residents, also those specialised services which are located outside the Study Area but which serve the patients of the study area are included, excluding primary care and social care services. The services inventory follows the ESMS/DESDE approach to service assessment [1,14,16]. It is composed of a user manual with all the instructions and the services inventory file to be compiled online. The compilation of the file will provide information on the general context of each service, its ecological setting, distribution and utilisation.

Five types of information are required for each service: Service basic information (4 items); Location and Geographical information about the service (3 items); Useful information and contacts (4 items); Service data (12 items); and Evaluator information [23]. The Service data information allows for a detailed description of each service through the collection of data on staff, availability and contacts. Definitions of technical terms are provided in the REFINEMENT glossary (www.refinementproject.eu).

**Figure 2.**
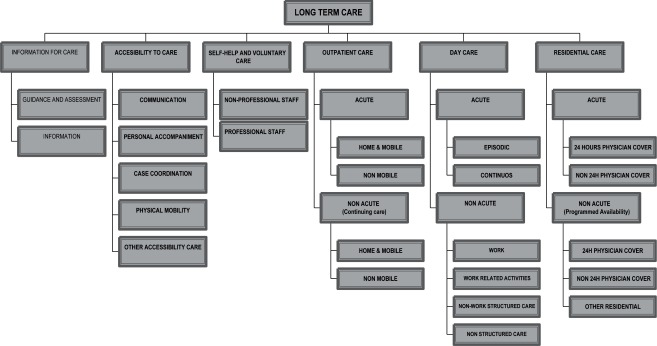
DESDE-LTC: Tree taxonomy of the main types of care [MTC] used for describing and coding ‘basic stable inputs of care’ [BSIC] as the minimum organisational unit of care delivery in mental health services.

The core of the Services Inventory is represented by a validated tool on its own, the “description and evaluation of services and directories in Europe for long-term care” (DESDE-LTC) that provides the information needed to complete the MHSI. DESDE-LTC is the extended version of the European service mapping schedule (ESMS-I) for the assessment of services in adult mental health care [22]. It increased the original 32 codes to 91 to allow for coding services in other sectors (education, employment, crime and justice), other specific groups (child and adolescents, elderly, drug addiction) and other health targets (disability and long-term chronic care). The feasibility, reliability and validity of both ESMS-I and DESDE-LTC have been previously described [1,16,22,24]. These instruments have been used in over 13 countries to provide regional, national and international comparisons of mental health systems and long-term care [16]. DESDE-LTC uses a tree system of the classification of services in a defined catchment area according to the main care structure/activity offered as well as their level of availability (section B) and utilisation (section C). The tree structure is shown in Figure 2.

### Geographical data

Geographical data together with the geocodification of services allow, through geographic information systems (GIS), the creation of an atlas of mental health maps. The atlas of mental health includes:

- Mapping of the main social and demographic indicators relevant to mental health care in the study areas by analysing available social and demographic data sets (e.g., deprivation index, dependency index, people living alone rate, unemployment rate). Information collected with the SES Index, the Services Inventory and the Geographical Data can be combined to provide a detailed description of both mapped services and the study areas. The SES Index provides information on the level of deprivation of each study area where services are located. Geographical Data can be combined with SES Index results to explore the relationship between social disadvantage and the quantity of services available. Analysing the social and demographic characteristics of service areas may reveal populations with unmet needs.
- Mapping of service provision (care availability, placement capacity, workforce capacity and accessibility) in each study area according to their main types of care. Rates and ratios on services availability (opening hours and days), and places (e.g., beds) describe the utilisation of the services of each study area. For instance, the ratio between the number of beds in mapped services and the adult population of each area multiplied by 100,000 is calculated and represented in the Atlas.
- Mapping of utilisation of mental health-related services in each study area by analysing available data sets (for instance, hospital discharge rates, mean length of stay, mean number of outpatient visits from the Mental Health Service Inventory). They also include rates and ratios of contacts with outpatient care and admissions to residential care beds and day care places.
- Mapping of the workforce. The staff was measured in Full Time Equivalents (FTE) and presented as multi-professional groups composed of different types of workers by BSICs.

### Procedure

**Figure 3.**
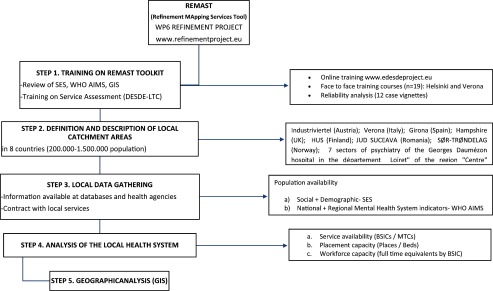
Flowchart describing the procedure followed in the research on financing systems’ effect on the quality of mental health care mapping services tool study.

The REMAST study was jointly coordinated by the University of Verona (Italy) and PSICOST and Loyola University (Spain) within the Refinement group. A formal partnership with official agencies was established with the Mental Health unit at the Department of Health in Catalonia (Spain) and with the Department of Health in Finland. Data collected in the eight REFINEMENT study areas refer to the years from 2008 to 2011. A flowchart describing the procedure followed in REMAST is included in Figure 3.

**Step 1 – Training on REMAST:** In the first Steering Committee in Verona (1–3 February 2011), different checklists and inventories and tools were reviewed to be used in the study (SES, WHO AIMS, GIS, etc.). Then, the DESDE-LTC approach to service assessment was presented and discussed by the REFINEMENT group. An online course and training material was made available to the researchers of the project partners (www.edesdeproject.eu). In addition, two face-to-face training courses were organised in Helsinki and Verona. In all, 19 researchers from all REFINEMENT centres except for Romania attended these courses. At the end of the training, researchers rated 12 case vignettes based on real services and showing 14 different MTCs. A multi-judge reliability analysis was made using the Cicchetti Index [25] to assess the level of inter-evaluator agreement, averaged across the 19 evaluators and the average level of paired agreement of each individual evaluator with each of the remaining 18 evaluators (Table 2).

**Step 2 – Definition and description of local catchment areas:** study areas of eight European countries were selected according to a rank of 200.000–1.500.000 inhabitants. The study areas selected are detailed in Figure 3.

**Step 3 – Local data gathering:** Identification of information available in databases and health agencies as well as direct contact with local services. The health-related services providing care for people with mental disorders were identified and described together with social and demographic characteristics and other relevant context information into an ad hoc database from the following sources: Mental Health Information Systems (e.g., social insurance and administrative databases, psychiatric case registers); data sets from institutes for statistics; Social Care questionnaires adapted from Health Care ones; interviews to both medical and administrative staff; previous reports; and official and reliable websites. The information registered in every service included: (a) Service basic information (e.g., name, type of service, description of governance); (b) Location and geographical information about the service (e.g., service of reference, service area); (c) Service data (e.g., opening days and hours, staffing, management, economic information, legal system, user profile, number of users, number of contacts or admissions, number of days in hospital or residential structure, number of available beds or places, links with other services); (d) Additional information (name of coder, date, number observations and problems registered in the data collection).

**Step 4 – Analysis of the local health system:** It included the analysis of three main areas (a) Service availability, basic stable inputs of care of the services were identified and coded according to their principal MTC using the DESDE-LTC classification system. (b) Placement capacity, aimed at counting the number of places and beds of the BSICs, and (c) Workforce capacity, that included “physicians”, “psychologists”, “nurses”, “social workers” and “occupational therapists” were counted using ‘Full Time Equivalents” (FTE) per every basic stable inputs of care and its principal MTC. The category “other workers” was applied to all those health workers whose job is not classified in the other five definitions. For instance, voluntary staff is included in this category. Missing data and non-congruencies were revised by the core group and a second review of the data was made in October 2012. Data cleaning, additional reviews and updates followed to create the final data set by September 2013 with a total of 748 observations. The territorial comparison was carried out using the principal MTC of every BSIC and the total number of MTCs identified in every area.

**Step 5 – Geographical analysis:** The geographical description of the catchment areas encompassed different geographical divisions (e.g., Census, Local Government, LHD Medicare Locals, Primary Care, Housing, etc.). The distribution of mental health services was described through geographical information systems (GIS) technology in all countries where integrated spatial data were available. In all REFINEMENT countries it was possible to get information on mental health services access, utilisation and the populations living in the services area.

Traditional methods to measure spatial accessibility to health care include provider-to-population ratios, travel time to nearest provider, average travel impedance to provider and gravity models that provide a measure that accounts for both proximity and availability. The spatial analysis in the REMAST mapping services tool makes use of catchment area analysis using drive time isochrones maps, for each of the eight REFINEMENT countries, to analyse the potential accessibility and ability to travel of the population to mental health services. The catchment area for a health care provider is the geographical area that contains the bulk of served population. Understanding jurisdictions and catchment areas is important for health care providers because it ties the client population to a particular area or set of communities. Adequacy, equity and efficiency can be analysed in every given area and related to the diversity of population health needs. Based on this analysis, it is possible to determine how many inhabitants live within one hour driving distance from a hospital or how many people have to drive longer than a given time to reach a hospital.

Another approach to estimate the spatial accessibility is the enhanced two-step floating catchment area (E2SFCA) whose application needs three main parameters: the supply, the demand and the computation of accessibility measures. The supply is represented by mental health services. All the addresses of the services mapped in the REMAST were geocoded using the Google Geocoding API (V3) and integrated into a GIS software. The demand is represented by the potential users of mental health services (represented by the population size and location data, obtained from GEOSTAT 1A). Finally, the distance between the supply and the demand locations as for the previous method has been calculated using the street networks dataset from OpenStreetMap. The travel time between each mental health services and population location can be calculated using the Origin-Destination cost matrix function of ArcGIS Network Analyst Extension.

Building on previous research [26] the E2SFCA method consists of applying weights to differentiate travel time zones, accounting for distance decay. In order to differentiate accessibility within a catchment, multiple travel time zones within each catchment are obtained using the ArcGIS Network Analyst and assigned with different weights according to the Gaussian function [27,28].

### Study endpoints

The primary endpoint for this study is to provide standardised rates of mental health service provision in selected study areas of 8 EU countries, according to four main domains: (a) Availability of main types of care (MTCs) in the study area as described by the total number of MTCs and the different types of MTCs available per 100,000 attended population; (b) Care placement capacity or rate of beds in residential BSICs and places assigned in structured day care to persons experiencing mental disorders in the study area; (c) Workforce capacity: rate of professional clinical staff measured in ‘Full Time Equivalents’ (FET) (total and by professional background: physicians, psychiatrists, nurses, psychologists); and (d) Geographical accessibility to Services and to BSICs as defined by its principal MTCs. The secondary endpoints to be considered are the quality indicators of relative efficiency and the feasibility of the REMAST tool for evidence-base policy, planning, priority setting and resource allocation.

### Ethical issues

The information provided on service availability and capacity did not require ethical approval in the countries of this study as they did not include data on individual patients. The information on service utilisation was mainly based on publicly available information. In Spain, a secondary analysis of available data sets was performed following authorisation and ethical approval from the regional Department of Health.

## Discussion

This context analysis of the implementation of local mental health systems follows an ecological approach. This project brings together practice, policy and research to inform mental health policy and priority setting in Europe. This project develops a new decision tool which is based on and integrates different instruments (WHO-AIMS 2.0, Verona SES Index, DESDE-LTC) and visualisation techniques (GIS) to produce local atlases of mental health care for the monitoring, reviewing and improving of the mental health systems in Europe. The methodological approach has been designed to overcome current problems of commensurability, semantic interoperability and representativeness in health service research, particularly in integrated care studies where services from different sectors have to be considered. The tree taxonomy approach followed in DESDE-LTC allows for inclusion of new codes when needed.

Results should illustrate the significance of spatial analysis in the allocation of health care services and facilitate the improvement of equitable access to health care. Policymakers and stakeholders should be informed of the location of services and be aware of the existence of shortage areas for a better provision of mental health care

Evidence-informed policy takes into account local factors, to assess a “country’s health and health systems challenges” and the development of “evidence-based responses to evolving challenges and opportunities, and to involve all relevant stakeholders” [29]. Within this context, the standard coding of service availability and capacity of care provision combined with geographical accessibility using GIS, support evidence informed policy by analysing and transforming complex data from various sources into visual maps that illustrate problem areas in the provision of services.

The ESMS/DESDE approach is an advance over previous attempts to code health services, although it has substantial limitations. It demands a considerable research effort as the basic sets of inputs (BSICs) at every specialised service available in a study area have to be identified and coded. In order to do that researchers need a high level of training which cannot be replaced by on-line material and short courses as it was previously found in the DESDE-LTC study [16]. Even though the DESDE-LTC training exercise included vignettes with incomplete information to provide examples of actual problems in data gathering, it is important to note that 28% of the 14 MTCs obtained poor agreement with the gold standard. Therefore monitoring of the data gathered at local level and the use of sensitivity analysis of the data obtained at the different settings is needed when DESDE-LTC is either used for routine analysis or as part of a broader analysis of health systems. It is also important to note that existing local differences on service delivery within a country does not allow us to extrapolate the information gathered in the selected study areas to the respective countries, so our study is illustrative and does not allow full country-to-country comparison. However differences across countries are higher than differences within countries, so the comparisons across selected areas may provide guidance that could be confirmed in broader studies. Full regional and national service mapping should be encouraged as main tools for health planning in the next future.

GIS is helpful for understanding a variety of health care issues such as defining hospital service areas, examining the effect of distance on access and disease patterns [30,31]. These visualisation tools for decision-making and quality assessment underline the disparities in accessing services among population groups. This system is in fact a structured system for the assessment of mental health care needs [30] suited to reach a broad array of audiences, including health planners, policy-makers, advocacy groups and an interested public. As a visual form of communicating health information, morbidity and care provision maps may bridge the gap between complex epidemiological presentations of statistics and the varied educational backgrounds represented by policy-makers, other stakeholders and users.

A number of new ethical issues arise when using spatial information and publishing local health atlases. First the availability of information on services provision and utilisation is a critical factor for health planning, equity and parity, and therefore the appropriateness of health planning in the absence of geographical information could be questioned. Second there is a huge disparity in the availability of service and geographical information across different countries and regions. Information accessibility is an integral part of care accessibility and it should be considered when analysing equity and parity. In the study areas in the eight partner countries we found large disparities in the availability of databases on the health and social resources, on their content for actual policy planning and on their overall quality. This was a major problem for estimating the timeline and for carrying out the mapping exercise. There is an urgent need for developing better standards of information on services, utilisation and quality at local, national and international level. The information on publicly funded services (availability, location, capacity and staff) should be accessible for researchers and for users. Due to its political implications, information on service research may be concealed to public access and scrutiny. The implications of not delivering all health service information are a matter of further ethical debate, in addition to their impact on quality of care. Third, the availability and access to care information could eventually compromise individual confidentiality even when pseudonymised data are used. Spatial analysis could provide detailed information not only of where care is provided but also on who receives it when household addresses are available. As an example spatial location of individual home care and individual residential services, and location of users of mental health services could be used to identify specific users below municipality or census area levels. The ethical requirements on the use of this information show huge disparities across the different countries and publishing recommendations guidelines should be developed.

## Conclusion

Standard coding and geospatial mapping of services has received little attention as a relevant determinant of mental health service utilisation [32] and in the analysis of integrated care. The outcomes of this project may facilitate cooperative work and knowledge transfer to public agencies and other institutions involved in mental health planning and provision, providing evidence to allocate resources at different jurisdiction levels, and will facilitate the assessment and the monitoring of integrated care at the local system level as well as the interventions implemented to enhance integrated care.

This project could also improve the information to users and society on the available resources for mental health care (via a user friendly public version of the local atlases), allowing a more ethical, transparent, and democratic participation in health issues. This study will also illustrate how atlases of integrated mental health care should be developed in the European Union and internationally. The techniques used in this project and the knowledge generated could eventually be transferred to the mapping of other areas of integrated care.

## Figures and Tables

**Table 1.**
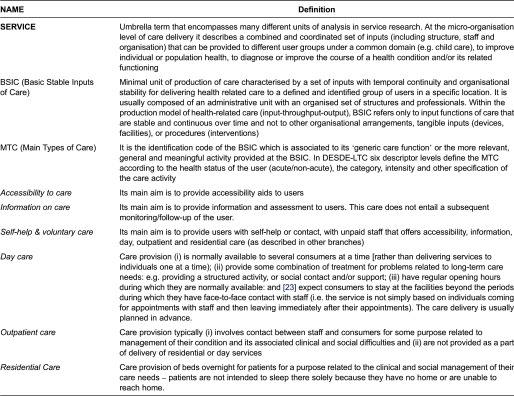
Main units of analysis in health service research at the micro-organisation level included at the DESDE-LTC classification system

**Table 2.**
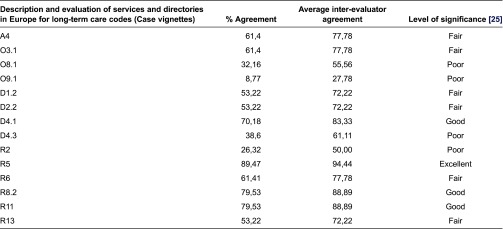
DESDE-LTC: Average agreement of 19 evaluators from 7 countries with the gold standard coding of main types of care based on 14 case vignettes and average level of paired agreement of each evaluator with each of the remaining 18 evaluators
